# How Employees’ Emotional Labor Promotes Perceived Service Quality: A Dual-Pathway Model

**DOI:** 10.3390/bs15111538

**Published:** 2025-11-11

**Authors:** Pengfei Cheng, Xu Zhao

**Affiliations:** School of Economics and Management, Xi’an University of Technology, Xi’an 710048, China; pfcheng@xaut.edu.cn

**Keywords:** emotional labor, service quality, customer positive affect, customer participation

## Abstract

Although service firms recognize the significance of frontline employees’ emotional labor in enhancing perceived service quality and sustaining competitive advantage, the theoretical mechanisms underlying this relationship remain insufficiently understood. Drawing on the Emotions as Social Information Model (EASI), this study proposed that frontline employees’ emotional labor influences customer perceived service quality through two distinct pathways: emotional contagion and inferential processing. Moreover, the relative strength of these two pathways is contingent upon customer involvement. Using dyadic data collected from frontline employees and customers in the banking sector, the results indicated the following: frontline employees’ different emotional labor strategies (deep acting and surface acting) exerted significant influence on perceived service quality through different pathways. Specifically, surface acting impacted service quality solely through emotional contagion process (via customers’ positive affect). Whereas deep acting influenced service quality through both emotional contagion (via customers’ positive affect) and inferential processing (via customer participation). Additionally, customer involvement moderated the relationship between deep acting and customer participation (strengthening the positive association), as well as the link between surface acting and customers’ positive affect (attenuating the negative association).

## 1. Introduction

In order to survive in a fiercely competitive environment, service organizations are increasingly raising expectations for frontline employees, who are key determinants of service evaluations (Madera et al., 2020). Especially in the high-contact service industry, customers evaluate service quality by interpreting frontline employees’ emotional displays and behaviors during service encounters (Y. Shin et al., 2022). Emotional labor, as conceptualized in Hochschild’s seminal work, refers to the process by which frontline employees regulate their emotional expressions to meet their job requirements (Hochschild, 1983). It is widely acknowledged by both academic researchers and practitioners that frontline employees’ emotional labor plays a critical role in shaping customers’ perceptions of service outcomes (Deng et al., 2017; I. Shin & Hur, 2020), such as customer service experience, satisfaction, and overall evaluations of service quality. However, as two different strategies employees can take to regulate their emotions, deep and surface acting show divergent effects on customers’ service evaluation. For instance, Andrzejewski and Mooney (2016) found that only genuine emotions displayed (deep acting) by frontline employees could lead to higher customer service evaluations. More recently, research conducted within the hospitality industry by C.-J. Wang (2020) demonstrated that deep acting and surface acting had divergent impacts on customers’ perceptions of service quality. Specifically, deep acting exerted positive effects on perceived service quality, whereas surface acting had a negative influence on service quality. Despite these insights, the underlying mechanisms through which emotional labor strategies differentially affect service quality remain unclear. Resolving this theoretical gap is essential for a more nuanced understanding of how employees’ emotional regulation translates into customer perceptions.

The Emotions as Social Information Model (EASI) provides a theoretical framework for understanding how observers interpret and utilize emotional displays as social cues (Van Kleef, 2009). According to the EASI model, interpersonal emotional influence occurs through two distinct mechanisms: (1) inferential processes, where observers derive meaning from emotional expressions to assess the expresser’s intentions or situational demands, and (2) affective reactions, wherein observers experience emotional responses that subsequently guide their behavior (Van Kleef, 2009). The EASI model has been widely applied to explain various organizational phenomena, including conflict resolution, leadership effectiveness, and team performance (Lee, 2018; W. Liu et al., 2017; Van Kleef & Côté, 2022). However, its application within the context of service interactions remains relatively underexplored. Only a limited number of studies, such as Z. Wang et al. (2017), have applied the EASI model to explore how frontline employees’ emotional expressions shape customer affect and behavioral intentions (Z. Wang et al., 2017). This paucity of research underscores a critical gap in understanding the mechanisms through which emotional labor strategies transmit social information during service encounters.

To address these research gaps, drawing on the EASI model, we propose a dual-pathway framework through which frontline employees’ emotional labor indirectly influences perceived service quality: (1) the affective reaction pathway, which mainly occurs through emotional contagion by introducing customers’ positive affect as a mediator; (2) the inferential pathway, which represents customers’ elaboration of emotional information and is operationalized by introducing customer participation as a mediator.

Furthermore, we also propose that customer involvement—which affects customer information-seeking and information-processing behaviors (Olk et al., 2021)—serves as a boundary condition for the indirect effects of frontline employees’ emotional labor on service quality. Specifically, we expect that customer involvement may amplify or attenuate the influence of deep and surface acting on service quality, depending on whether the underlying mechanism is contagion (affective-driven) or inferential (cognition-driven).

By delineating these mediating pathways and boundary conditions, this study unpacks the “black box” linking emotional labor to service quality, thereby advancing theoretical understanding in this domain. Practically, our findings offer managers actionable insights into how emotional labor strategies differentially affect service outcomes, enabling more targeted interventions to enhance service delivery.

## 2. Theoretical Background and Hypothesis Development

### 2.1. Emotional Labor

Emotional labor refers to a series of behaviors performed by frontline employees to comply with organizations’ expectations by regulating their emotions during service encounters (Ashforth & Humphrey, 1993). Prior research has proposed two distinct strategies to regulate emotions: surface and deep acting (Grandey & Melloy, 2017). Surface acting occurs when frontline employees camouflage external emotional displays to conform with organizational display rules (Scott & Barnes, 2011). Deep acting, in contrast, refers to a form of emotional regulation performed with greater sincerity. Frontline employees who engage in deep acting try to adjust inner emotional experiences to be consistent with the mandated emotional display rules. Many previous studies emphasized the negative consequences of surface acting, such as reduced employee well-being (Yao & Gao, 2021). In contrast, the outcomes of deep acting tend to be desirable. For example, deep acting is positively associated with performance outcomes, especially customer satisfaction (Gabriel et al., 2023; Groth & Esmaeilikia, 2023).

Regarding the interactive nature of service, a stream of research has focused on the mechanism by which frontline employees’ emotional labor exerts effects on a customer’s service evaluation. From the employee perspective, Hur et al. (2015) pointed out that emotional labor influences employee job satisfaction and, in turn, predicts customer satisfaction (Hur et al., 2015). The recent work by Wu et al. (2024) indicated that emotional labor influenced service quality through employee work fatigue (Wu et al., 2024). In contrast, there were more studies exploring the mechanism from a customer perspective. For example, Lechner and Mathmann (2021) found employees’ inauthentic display of emotions would lead to inferred deception and, in turn, undermine the service performance (Lechner & Mathmann, 2021). Chaker et al. (2021) noticed customers’ perception of an employee’s emotional labor would trigger different response behaviors (revenge, avoidance, and forgiveness) (Chaker et al., 2021). In addition to customers’ cognitive or behavioral responses, Gong et al. (2020) found that emotional labor would influence customers’ affect through contagion and further influence service quality (Gong et al., 2020). Therefore, both the cognitive and affective mechanisms should be considered simultaneously.

### 2.2. Service Quality

As one of the most popular concepts in the service marketing field, the perceived service quality refers to individuals’ judgment about service production and delivery, which is “formed by customers during their service experience” (Dlačić et al., 2014; Pugh, 2001). Service quality includes two distinct dimensions (Ha & Jang, 2010). Technical quality emphasizes the tangible aspect of the service, such as the accuracy, reliability, and effectiveness of the service provided (Gallan et al., 2013). In contrast, functional quality focuses on the process of how service is delivered. Therefore, customers’ interaction experiences with service employees directly shape their perception of functional quality (De Keyser & Lariviere, 2014). Both dimensions are crucial for delivering high-quality service (Gronroos, 1993).

Frontline employees, as representatives of service firms, are a critical factor impacting service quality (Fan et al., 2022; Lin et al., 2021). A lot of studies have provided evidence to support that employees’ deep acting leads to high service performance (Chi & Grandey, 2019; C.-J. Wang, 2020). However, there were inconsistent findings on the relationship between frontline employees’ emotional labor and service quality. For example, Huang and Lin (2021) found that frontline employees’ emotional labor (either deep acting or surface acting) did not have significant effects on customers’ perceived service quality (Huang & Lin, 2021). Cheshin et al. (2018) pointed that employees’ emotional displays could be interpreted as inappropriate and inauthentic and could lead to reduced trust in the employees (Cheshin et al., 2018). Therefore, the boundary conditions of the relationship between frontline employees’ emotional labor and service quality should be explored.

### 2.3. A Dual Pathway for Emotional Labor to Influence Perceived Service Quality

The EASI model posits that emotions serve as critical social cues in interpersonal transactions. Observers infer the expresser’s underlying motivations by using these emotional cues and, subsequently, respond to them (Van Kleef, 2009). According to the EASI model, observers process others’ emotional information through two distinct pathways, which guide their subsequent responses (Deng et al., 2020). The emotional contagion pathway refers to a process by which individuals automatically imitate and synchronize facial expressions, actions and vocalizations with others, leading to emotional convergence (Herrando et al., 2022). This pathway represents the fundamental aspect of social bonding and empathy, enabling individuals to resonate emotionally with others. In contrast, the inferential pathway involves a cognitive process wherein individuals use emotional displays to deduce the underlying feelings, intentions, and motivations of the expressers (Erle et al., 2022). Unlike the automatic and unconscious nature of the emotional contagion pathway, the inferential pathway requires observers to engage in deliberate and effortful cognitive processing to interpret the true emotional states and motivations of expressers (Van Kleef, 2009). Following the EASI model, customers, as observers of frontline employees’ emotional displays, are expected to utilize these two pathways to interpret frontline employees’ underlying motivations and subsequently evaluate the service quality.

#### 2.3.1. The Emotional Contagion Pathway of Emotional Labor on Perceived Service Quality

According to the emotional contagion theory, individuals’ emotional reactions can be evoked by others’ emotional displays (Hatfield et al., 2014; Herrando & Constantinides, 2021). During this process, individuals automatically mimic and unconsciously synchronize other’s emotional states and behaviors (Kong, 2022; Norscia et al., 2020). There are two distinct contagion process: primitive contagion and deliberate contagion. Primitive contagion occurs when individuals unconsciously and automatically synchronize with the emotions of others without devoting much cognitive effort to judging the authenticity (Hatfield et al., 1992). Therefore, even when frontline employees display fake positive emotions through surface acting, customers can still be affected and experience similar positive emotions through primitive contagion. Following this viewpoint, frontline employees’ surface acting is expected to exert a positive influence on a customer’s positive affect.

In contrast, deliberate contagion involves the intentional transfer of emotions through verbal, nonverbal, or behavioral cues. In service interactions, frontline employees engage in deep acting through deliberately regulating their inner feelings and expressing positive emotions, aiming to create an excellent service experience for customers. Thus, deep acting is also expected to foster a customer’s positive affect—a proposition supported by empirical evidence linking employees’ positive displays to customers’ positive affect (Pugh, 2001). Consequently, regardless of whether the positive emotions displayed by frontline employees are genuine (deep acting) or inauthentic (surface acting), both emotional labor strategies can induce positive customer affect through the emotional contagion pathway. We propose the following:

**H1.**  *Frontline employees’ (a) deep acting and (b) surface acting are positively related to a customer’s positive affect.*

The quality of interactions between frontline employees and customers constitutes a critical factor in service quality evaluation. Especially in high-contact service settings, a customer’s positive affect—a central aspect of their service experience—serves as a pivotal determinant of their service evaluations (Zeithaml et al., 1988). Research on affect infusion suggested that customers’ service evaluation was influenced by their affective states (Forgas, 1995). Consequently, a positive affect can diminish customers’ critical thinking, leading to enhanced perceptions of service quality (Barger & Grandey, 2006; Rocklage & Fazio, 2020). We propose the following:

**H2.**  *Customers’ positive affect is positively related to (a) technical service quality and (b) functional service quality.*

#### 2.3.2. The Emotional Inferential Pathway of Emotional Labor on Perceived Service Quality

The EASI model posits that the inferential pathway leads observers to interpret an expresser’s motivations through their emotional displays (Van Kleef, 2009). This cognitively effortful process is pivotal in forming behavioral responses (Bruder et al., 2021; Erle et al., 2022). In service encounters, the authenticity of frontline employees’ emotional display is the critical trigger for this pathway. When customers perceive frontline employees’ positive emotions as authentic (deep acting), they have positive emotions and further make appreciate behavioral reactions (Gong et al., 2020). On the contrary, if customers consider employees’ emotions as faked (surface acting), they are likely to question frontline employees’ underlying motivation, which damages trust (X. Y. Liu et al., 2019). Therefore, the inferential pathway is uniquely activated by deep acting rather than surface acting.

Customer participation refers to the extent of customers’ resource investments in the co-production and delivery of services (Morgan et al., 2018). This involvement necessitates the investment of additional resources, such as time, energy, and personal effort, from the customer (M. Li & Hsu, 2017). Conceptually, customer participation constitutes a form of elaborate cognitive processing that is primarily activated during the inferential pathway of emotional interpretation. Therefore, we propose that in service interactions, frontline employees’ deep acting can stimulate customer participation, and this effect is predominantly mediated through the emotional inferential pathway rather than the contagion pathway.

**H3.**  *Deep acting is positively related to customer participation.*

The inseparability of service implies that its quality is jointly shaped by employee and customer actions (Rather & Camilleri, 2019). Customer participation serves as a critical mechanism in this co-creation process. From the service firm’s perspective, it provides vital information for customizing offerings and enhancing technical quality (Gallan et al., 2013). From the customer’s perspective, it fosters a more informed and realistic understanding of the service, mitigating expectation gaps (Barari et al., 2021). Moreover, participation directly enhances the functional quality by transforming the interaction dynamics. When customers engage in co-creation (Kumar & Pansari, 2016), they gain a greater sense of control, which enriches the service experience (Auh et al., 2019). In addition, customer participation also fosters communication and strengthens relational bonds between customer and frontline employees (Delpechitre et al., 2018). Thus, customer participation simultaneously improves both the technical and functional dimensions of service quality. We propose the following:

**H4.**  *Customer participation is positively related to (a) technical service quality and (b) functional service quality.*

### 2.4. Moderating Effect of Customer Involvement

Customer involvement is defined as a customer’s perceived personal relevance of an object or decision, based on their inherent needs, values, and interests (Cui & Wu, 2016). Different from customer participation, which emphasizes specific actions (e.g., sharing information) in service delivery, customer involvement is about the customer “caring” and having a mental, emotional, or psychological connection with brands or organizations. As a kind of psychological state variable, customer involvement has been identified as a critical motivational factor that can influence customers’ purchase decisions, information-seeking behaviors, and way of information processing (C. M. Cheung et al., 2012; Y. Li et al., 2019). According to the Information-processing Theory (Hann et al., 2007), highly involved customers are characterized by elevated cognitive needs, prompting a more effortful and analytical processing of information to inform their judgments. Consequently, we argue that customers with high involvement are more motivated to expend cognitive effort in interpreting employees’ emotional displays, thereby engaging more actively in the emotional inferential pathway. We propose the following:

**H5.**  *The positive influence of deep acting on customer participation is stronger (weaker) when customer involvement is high (low).*

**H6.**  *The positive influence of surface acting on customers’ positive affect is weaker (stronger) when customer involvement is high (low).*

**Figure 1:**
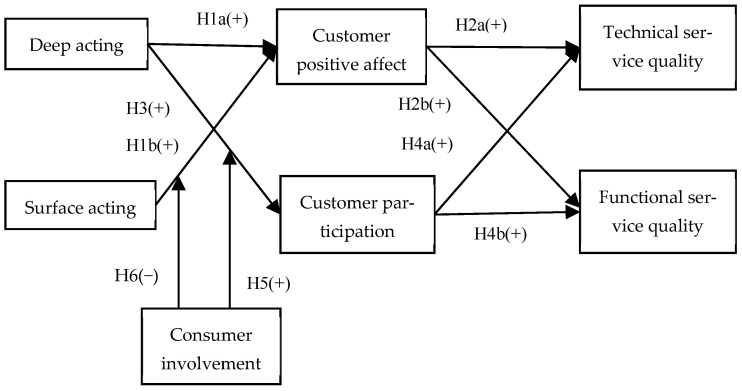
The conceptual model.

All of the aforementioned research hypotheses constitute the conceptual model of this study (as shown in Figure 1). 

## 3. Method

### 3.1. Sample and Procedure

To empirically test the conceptual model, data were collected in the retail banking industry, a context characterized by intensive employee–customer interactions. In this setting, frontline employees are typically required to manage their emotional expressions, which plays a vital role in determining the quality of the service. Dyadic data were collected on-site from both frontline employee and customers in ten retail banks located in northwestern China. Before the data collection, the aim and the procedures of the survey were introduced to frontline employees carefully during the morning shift meetings. Frontline employees who consented to participate this survey were then provided with a questionnaire. To capture the dynamic nature of emotional labor, frontline employees were asked to report the extent of the emotional labor they had just performed upon completing a service interaction. When customers accepted the invitation of participating in the survey, they were asked to report their level of positive affect, participation, involvement, and perceived service quality. Each frontline employee questionnaire was matched with their corresponding customer questionnaire to form a dyadic sample. Out of 450 distributed dyadic questionnaires, 371 were returned. After removing 17 incomplete responses, 354 were usable, for a response rate of 78.7%.

### 3.2. Measures

All core constructs (All items were presented in Appendix A) were measured by seven-point Likert scales ranging from 1 (extremely disagree) to 7 (extremely agree). Specifically, frontline employees reported their deep and surface acting using two 3-item scales developed by Brotheridge and Lee (2003). The sample items of deep acting and surface acting were “Make an effort to actually feel the emotions that I need to display to customers” and “Pretend to have emotions that I don’t really have”, respectively. Customer participation was reported by customers using a scale with five items developed by Chan et al. (2010). The sample item was “I have a high level of participation in the service process”. Customers reported their positive affect using a 6-item scale from Pugh (2001), originally developed by Brief et al. (1988). The sample item was “I felt excited during the service”. With respect to service quality, we adopted two 3-item scales from Gallan et al. (2013) to capture the two key dimensions: functional and technical quality, respectively. The sample items of functional and technical quality were “The employee treated me with respect” and” The employee is quite skilled in his/her job”. Customer involvement was measured using 4 items by M. F. Y. Cheung and To (2011). The sample item was “My banking service with this bank is very important to me”. Control variables included gender, age, income, and education. The demographics information of the sample is presented in Table 1.

### 3.3. Descriptive Statistics, Reliability, and Validity

Before testing the hypotheses, the validity and reliability of the measurement scales were assessed. The means, standard deviations, correlation coefficients, and Cronbach alpha coefficients for all constructs are shown in Table 2. The Cronbach alpha coefficients ranged from 0.817 to 0.918, exceeding the cut-off value of 0.70. Therefore, the reliability of all constructs was acceptable.

Regarding validity, we performed confirmatory factor analyses (CFAs) by restricting each item to only load on its theoretically specified factor. The results (as shown in Table 3) of the CFAs indicated that the hypothesized 7-factor measurement model fitted the data better than any other alternative measurement models with 5 to 1 factors, suggesting the acceptable validity of all measures.

Further, although the dyadic data could reduce the risk of common method variance, the data of dependence constructs (functional and technical quality), mediators (customers’ positive affect and customer participation), and moderator (customer involvement) were collected through customers’ self-reporting. Therefore, Harman’s one-factor test was performed as recommended by Podsakoff and Organ (1986). As we expected, the first factor accounted for 29.49% of the total variance, ruling out the presence of common method variance.

## 4. Results

### 4.1. Test of the Mediating Effects

To examine the mediation effects of customer participation and a customer’s positive affect on the link between emotional labor and technical service quality, we ran several regression analyses by using the PROCESS macro (Model 4) developed by Hayes (2018). First, we tested the effects of deep acting on the technical service quality (TSQ) through both customer participation and a customer‘s positive affect. The results of M-1 in Table 4 indicated that deep acting exerted positive and significant (β = 0.443, *p* < 0.001) influences on a customer’s positive affect. H1a was supported. Similarly, the results of M-2 indicated that the effect of deep acting on customer participation was significant and positive (β = 0.321, *p* < 0.001), providing support for H3. Subsequently, we regressed technical service quality (TSQ) on the independent variables (deep acting) and mediators (a customer’s positive affect and customer participation) simultaneously. The results of M-3 showed that: under the control of mediators, (a) the effect of deep acting on technical service quality became non-significant; (b) both customers’ positive affect (β = 0.349, *p* < 0.001) and customer participation (β = 0.512, *p* < 0.001) had significant positive effects on technical service quality, thereby supporting H2a and H4a. These results indicated that the influence of deep acting on technical service quality was fully mediated by both customer participation and a customer’s positive affect. Bootstrap analyses further confirmed these findings. As shown in Table 5, neither the 95 percent confidence intervals of the indirect path from deep acting to technical service quality (TSQ) via customer participation (0.098, 0.245) nor via customers’ positive affect (0.095, 0.220) include zero. Therefore, both the indirect paths from deep acting to technical service quality (TSQ) via customer participation and customers’ positive affect were significant.

To examine the mediation effects of customer participation and customers’ positive affect on the link between deep acting and functional service quality, we regressed the functional service quality (FSQ) on deep acting and mediators, simultaneously. The coefficients of M-4 in Table 4 indicated that deep acting (β = 0.221, *p* < 0.001) had a significant positive direct influence on functional service quality (FSQ). Furthermore, both customers’ positive affect (β = 0.291, *p* < 0.001) and customer participation (β = 0.204, *p* < 0.001) were significant positive predictors of functional service quality (FSQ), providing support for H2b and H4b. The results (as shown in Table 5) of bootstrap analyses also supported the existence of parallel mediators. Neither the 95 percent confidence intervals of the indirect path from deep acting to functional service quality (FSQ) via customer participation (0.024, 0.118) nor via customers’ positive affect (0.063, 0.203) include zero. Therefore, both the indirect paths from deep acting to functional service quality (FSQ) via customer participation and customers’ positive affect were significant.

To test the influence of surface acting on the service quality through customers’ positive affect, the results of M-5 in Table 4 indicated that surface acting (β = 0.273, *p* < 0.001) had a significant positive influence on customers’ positive affect, supporting H1b. Furthermore, the coefficients of M-6 in Table 4 indicated that the link between surface acting and technical service quality (TSQ) was not significant, while customers’ positive affect had a positive and significant (β = 0.530, *p* < 0.01) influence on the technical service quality (TSQ). These results suggested that the influence of surface acting on technical service quality (TSQ) was fully mediated by customers’ positive affect. The result of bootstrap analyses in Table 5 (0.061, 0.189) also confirmed the mediating effect of customers’ positive affect on the link of surface acting to technical service quality.

Considering the indirect effect of surface acting on functional service quality (FSQ), the results of M-7 indicated that both surface acting (β = 0.145, *p* < 0.001) and customers’ positive affect (β = 0.366, *p* < 0.001) had significant positive influence on functional service quality. These results indicated that the influence of surface acting on functional service quality (FSQ) was partially mediated by a customer’s positive affect, which was also confirmed by the result of the bootstrap analyses shown in Table 5 (0.051, 0.159).

### 4.2. Test of the Moderating Effects

To test the moderating effect of customer involvement on the relationship between deep acting and customer participation, a regression analysis was conducted using the PROCESS macro (Model 7) (Hayes, 2018). To avoid multicollinearity issues, we centered the core variables prior to regression testing. The regression coefficients of M-8 (in Table 6) indicated that the main effects of both deep acting (β = 0.363, *p* < 0.001) and customer involvement (β = −0.404, *p* < 0.01) on customer participation were significant. Furthermore, the interaction item between deep acting and customer involvement (DA*CI) was positive and significant (β = 0.216, *p* < 0.001). In addition, the R^2^ change was significant (ΔR^2^ = 0.136, *p* < 0.001). These results indicated that customer involvement could strengthen the positive effect of deep acting on customer participation. Therefore, H5 was supported.

It was worth noting that the moderator customer involvement had negative and significant (β = −0.404, *p* < 0.01) effect on customer participation. This counter-intuitive finding makes sense when we consider the unique context of banking services. Dong et al. (2008) pointed out that, in situations of high perceived failure, a highly involved customer may prefer the firm to take full control of the recovery (Dong et al., 2008). Given the character of the high perceived risks in banking service, highly involved customers care so much about the risks and outcomes of financial services that they need to be absolutely sure they are being handled correctly. Therefore, customers who are highly involved hesitate to participate in banking transactions.

**Figure 2:**
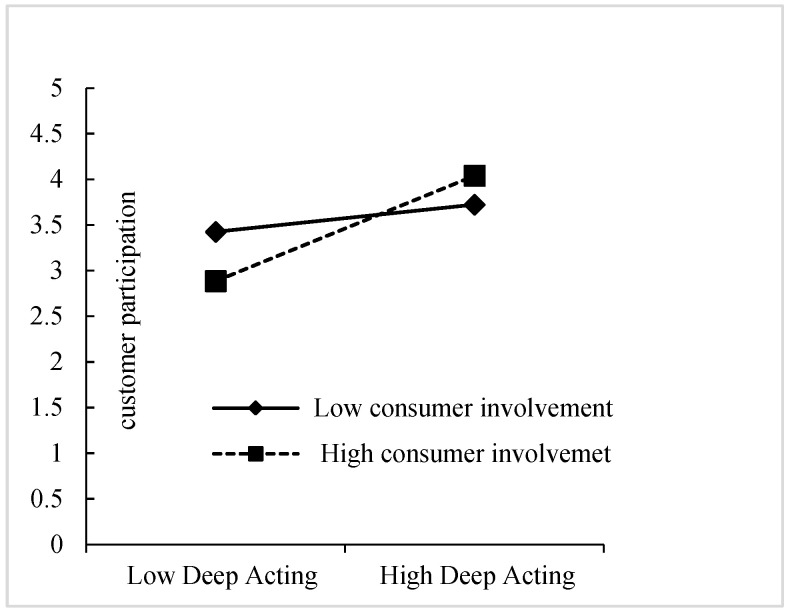
Moderating effect of customer involvement on deep acting–customer participation.

We visualized the moderating effect by plotting the simple slopes of the deep acting–customer participation relationship at high (+1SD) and low (−1SD) levels of customer involvement. As illustrated in Figure 2, the relationship between deep acting and customer participation was positive and stronger for highly involved customers. In addition, we also tested the significance of the moderated mediating effect. The bootstrap results (as shown in Table 7) indicated that, for customers with high involvement (+1SD), the indirect effects of deep acting on both the technical service quality (TSQ) (0.224, 0.477) and functional service quality (FSQ) (0.037, 0.219) through customer participation were significant, as the confidence intervals did not include zero. However, for customers with low involvement (−1SD), neither the indirect effect of deep acting on technical service quality (TSQ) (−0.055, 0.306) nor functional service quality (FSQ) (−0.019, 0.042) through customer participation were significant.

For the moderating effect of customer involvement on the relationship between surface acting and customers’ positive affect, the coefficients of M-9 shown that both surface acting (β = 0.494, *p* < 0.001) and customer involvement (β = 0.559, *p* < 0.001) had significant and positive influences on a customer’s positive affect. The regression coefficient of the interaction item between surface acting and customer involvement (SA*CI) was negative and significant (β = −0.062, *p* < 0.05). The R^2^ changes were also significant (ΔR^2^ = 0.011, *p* < 0.05). Thus, H6 was supported.

**Figure 3:**
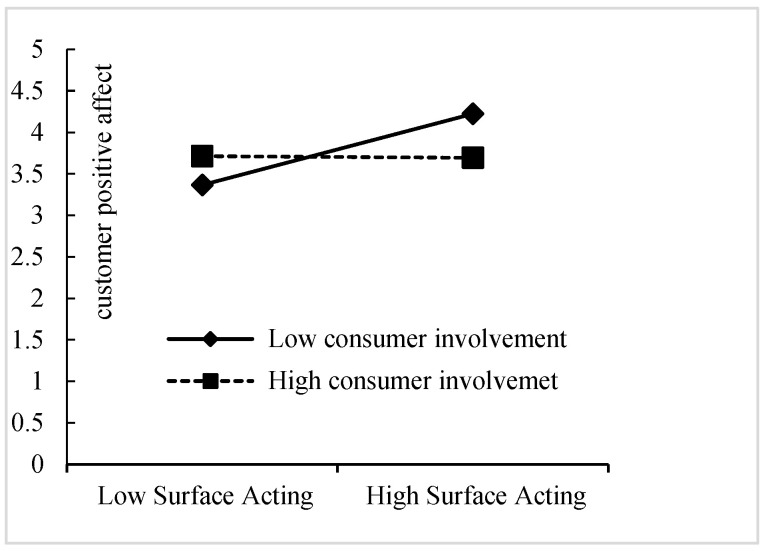
Moderating effect of customer involvement on surface acting–customers’ positive affect.

We plotted the simple slopes of surface acting on customers’ positive affect at high (+1SD) and low (−1SD) levels of customer involvement. As shown in Figure 3, for customers with low involvement (−1SD), frontline employees’ high surface acting fostered higher positive affect than those with high involvement (+1SD). The bootstrap results (as shown in Table 7) of the moderated mediating effects test showed that, for customers with low involvement (−1SD), the indirect effects of surface acting on both technical service quality (TSQ) (0.074, 0.229) and functional service quality (FSQ) (0.066, 0.189) through customers’ positive affect were significant. In contrast, for customers with high involvement (+1SD), neither the indirect effect of surface acting on technical service quality (TSQ) (−0.014, 0.137) nor functional service quality (FSQ) (−0.010, 0.120) through customers’ positive affect were significant.

## 5. Discussion

Drawing upon the EASI model, this study proposes and tests a dual-pathway framework through which emotional labor influences service quality, with customer participation (inferential pathway) and customers’ positive affect (emotional contagion pathway) serving as mediators and customer involvement as a key boundary condition.

The findings reveal that deep acting enhances service quality through the emotional contagion pathway and the inferential pathway. Specifically, its effect on technical service quality is fully mediated by both customers’ positive affect and customer participation. In contrast, its indirect effect on functional service quality is partially mediated by customers’ positive affect and customer participation. Deep acting also retains a significant direct influence on functional service quality.

Surface acting, however, influences service quality primarily through the emotional contagion pathway alone. Its effect on technical service quality is fully mediated by customers’ positive affect, while its impact on functional service quality is partially mediated by the same mechanism.

Furthermore, customer involvement moderates these indirect relationships. For highly involved customers, deep acting influences service quality mainly through the inferential pathway (customer participation). Conversely, for customers with low involvement, the effects of emotional labor are channeled primarily through the emotional contagion pathway (customers’ positive affect). The findings indicated that, for customers with different level of involvement, frontline employees’ distinct emotional labor strategies (deep acting and surface acting) exert influence on service quality through different pathways.

### 5.1. Theoretical Contributions

Both theoretical and practical perspectives recognize that frontline employees’ display of positive emotions enhances service experiences and evaluations (Rocklage & Fazio, 2020; Zhan et al., 2021). However, the mechanisms linking specific emotional labor strategies to service quality remain underexplored (Lechner & Mathmann, 2021). To address this gap, the current study draws on the EASI model to propose parallel mediating pathways—the emotional contagion pathway (via customers’ positive affect) and the inferential pathway (via customer participation) drawing on EASI model, we proposed that emotional contagion pathway (customers’ positive affect) and the inferential pathway (customer participation)—connecting employees’ emotional labor to perceived service quality. Our findings empirically identify two distinct pathways through which different emotional labor strategies influence service quality. This directly responds to recent calls in the literature to uncover the psychological processes by which customers internalize and interpret employees’ emotional displays, which in turn shape their service quality perceptions (Subramony et al., 2021). Moreover, the findings of this study answer calls raised by Van Kleef and Côté (2022) to investigate the mechanisms driving behavioral responses to emotional expressions, thereby extending the application of the EASI model in service contexts (Van Kleef & Côté, 2022).

Second, this study contributes to emotional labor theory by delineating the distinct mechanisms through which different emotional labor strategies influence service evaluations. While prior research, such as Gong et al. (2020), has suggested that frontline employees’ emotional labor would affect customer loyalty through affective reactions and cognitive appraisals (Gong et al., 2020), the differential pathways for deep and surface acting have received little attention. Our findings clarify that deep acting simultaneously triggers both the emotional contagion and inferential processes in customers. In contrast, surface acting primarily activates only the emotional contagion pathway. Thus, this study provides a more nuanced understanding of how customers utilize their perceptions of employees’ emotional expressions to form service evaluations.

Finally, this study contributes to the Emotions as Social Information (EASI) literature by identifying a critical boundary condition for this model in service interactions context: customer involvement. In doing so, we directly respond to the call by Van Kleef and Côté (2022) to extend and test the EASI model in diverse organizational settings (Van Kleef & Côté, 2022). Our results demonstrate that customers with high involvement are more likely to utilize the inferential pathway, carefully interpreting the meaning behind employees’ emotional displays. Conversely, customers with low involvement are more susceptible to the emotional contagion pathway, reacting automatically to the affective cues. These findings not only validate the EASI model in a service context but also provide clear evidence for the contingent nature of its underlying processes.

### 5.2. Managerial Implications

The findings of this study shed light on how service firms could improve service quality. First, managers should realize the vital role of frontline employees’ emotional labor in shaping customer’s perception of service quality. To foster more effective emotional regulation, firms can implement training techniques such as cognitive reappraisal and attention deployment. Cognitive reappraisal training can help frontline employees to reframe stressful situations. For instance, frontline employees could view dysfunctional customers not as a personal attack but as someone facing a genuine problem seeking help. These methods can guide frontline employees toward adopting deep acting—genuinely aligning their feelings with displayed emotions—rather than relying on surface acting during service encounters (Kang & Jang, 2022). Furthermore, managers can take specific leadership to support frontline customers to performing deep acting. For example, prior study has indicated that empowering leadership could provide frontline employees with adequate job resources by giving employees the discretion to solve common problems without excessive escalation (Cheng et al., 2025). In the banking service context, managers can provide guidelines instead of rigid scripts, allowing frontline employees to adapt their emotional expression to the specific context of the interaction.

Second, it is crucial for frontline employees to be aware of the customers’ level of involvement. When serving highly involved customers, frontline employees should prioritize deep acting to convey authentic positive emotions, as these customers are more likely to scrutinize the sincerity of the interaction (Kim & Park, 2023). Furthermore, frontline employees should take measures to facilitate high-quality customer participation. For example, the bank frontline employees can provide financial choices and encourage customers to make trade-offs together. Conversely, for customers with low involvement, who are less sensitive to the authenticity of emotional displays, frontline employees can allocate their cognitive resources more efficiently. In these cases, the focus can shift toward other critical aspects of the service, such as maximizing productivity (Bellet et al., 2024) and ensuring operational accuracy (Soemah, 2023).

## 6. Limitations and Future Directions

Several limitations of this study should be considered. First, the data of this study were collected exclusively from the retail banking industry in a specific region of China, which may limit the generalizability of the findings. Future studies should seek to replicate this research across diverse service sectors (e.g., hospitality, retail) and cultural contexts to enhance the external validity of this proposed model. Second, according to the EASI model, individuals’ ability to determine how to process others’ emotional cues depends on their competence in information processing. It is, therefore, necessary to consider new moderators of this mediation. Emotional intelligence (EI), which refers to the ability to recognize, understand, manage, and influence emotions—both in yourself and others (Prentice et al., 2020), should be considered as a new moderator in the future. Finally, the current study tested a dual-pathway framework through which emotional labor influences service quality. However, the potential interaction effects of these two paths were not considered. Future studies could explore whether and how these two paths interact with each other.

## 7. Conclusions

The findings of this study indicate that frontline employees’ emotional labor influences perceived service quality through two distinct psychological pathways, as outlined by the EASI model. Specifically, deep acting enhances service quality through both the emotional contagion pathway (by boosting customers’ positive affect) and the inferential pathway (by encouraging customer participation), whereas surface acting influences service quality only through the emotional contagion pathway (by boosting customers’ positive affect) and not through the inferential pathway. Furthermore, the study identifies customer involvement as a critical boundary condition of these mediation effects. For customers with high involvement, the inferential pathway (via customer participation) is more effective for deep acting. In contrast, for customers with low involvement, the emotional contagion pathway (via customers’ positive affect) is the primary mechanism through which both emotional labor strategies operate. In summary, this study successfully unpacks the “black box” linking emotional labor to service quality, demonstrating that the effectiveness of different emotional strategies depends on the mediating pathway they activate and is contingent upon the customer’s level of involvement.

## Figures and Tables

**Table 1:** Demographics information.

Demographics	Sample Size: 354	
Gender	Female	198
	Male	156
Age	<20	4
	20–29	215
	30–39	75
	40–49	39
	50–59	9
	60–69	8
	>70	4
Income (RMB/M)	<4000	92
	4000–5999	96
	6000–9999	112
	10,000–14,999	28
	15,000–19,999	22
	>20,000	4
Education	Below high school	2
	High school	49
	Junior college	104
	Undergraduate	164
	Postgraduate	35

**Table 2:** Descriptive statistics.

	Means	SD	1	2	3	4	5	6	7
1. DA	4.414	1.406	(0.899)						
2. SA	4.263	1.299	0.274 **	(0.817)					
3. CPA	4.261	1.347	0.454 **	0.298 **	(0.918)				
4. CP	4.775	1.354	0.366 **	0.140 **	0.02	(0.909)			
5. CI	4.225	1.48	0.309 **	0.105 *	0.380 **	0.07	(0.895)		
6. TSQ	4.766	1.582	0.360 **	0.021	0.337 **	0.460 **	−0.170 **	(0.890)	
7. FSQ	4.435	1.386	0.419 **	0.237 **	0.385 **	0.276 **	−0.160 **	0.328 **	(0.871)

Note: The Cronbach alpha coefficients are presented in parentheses. DA = deep acting, CP = customer participation, CPA = customer positive affect, SA = Surface acting, CI = consumer involvement, TSQ = technical service quality, FSQ = functional service quality. * *p* < 0.05; ** *p* < 0.01.

**Table 3:** Results of confirmatory factor analysis.

CFAs	Χ^2^	df	Χ^2^/df	RMSEA	SRMR	GFI	IFI	CFI	TLI
7-factor	814.25	303	2.687	0.069	0.0485	0.850	0.923	0.922	0.910
5-factor	1707.043	314	5.436	0.112	0.0994	0.718	0.789	0.787	0.762
3-factor	3035.951	321	9.458	0.155	0.1753	0.572	0.588	0.586	0.547
1-factor	4589.219	324	14.164	0.193	0.1773	0.413	0.352	0.349	0.295

**Table 4:** Mediating effects test.

Independent Variables		M-1	M-2	M-3	M-4	M-5	M-6	M-7
		CPA	CP	TSQ	FSQ	CPA	TSQ	FSQ
Control variables	Gender	0.017	0.085	−0.025	−0.067	0.164	0.124	0.036
	Age	−0.225 **	0.118	−0.060	−0.036	−0.177 *	−0.042	0.029
	Income	−0.030	0.142 *	0.019	0.019	0.034	0.181 *	0.089
	Education	−0.017	−0.147	0.091	0.067	−0.030	−0.015	0.030
DA		0.443 ***	0.321 ***	0.073	0.221 ***	--	--	--
SA		--	--	--	--	0.273 ***	−0.124	0.145 **
CP		--	--	0.512 ***	0.204 ***	--	--	--
CPA		--	--	0.349 ***	0.291 ***	--	0.429 ***	0.366 ***
R^2^		0.242 ***	0.174 ***	0.326 ***	0.263 ***	0.105 ***	0.136 ***	0.177 ***

Note: DA = deep acting, CP = customer participation, CPA = customer positive affect, SA = Surface acting, CI = consumer involvement, TSQ = technical service quality, FSQ = functional service quality. * *p* < 0.05; ** *p* < 0.01; *** *p* < 0.001.

**Table 5:** Direct and indirect effects of emotional labor on service quality.

Path			Effect	SE	95% Confidence Interval	
					Lower	Upper
DA → TSQ	Direct effects		0.073	0.062	−0.049	0.195
	Indirect effects	DA-CP-TSQ	0.164	0.037	0.098	0.245
		DA-CPA-TSQ	0.154	0.032	0.095	0.220
DA → FSQ	Direct effects		0.221	0.057	0.109	0.332
	Indirect effects	DA-CP-FSQ	0.065	0.024	0.024	0.118
		DA-CPA-FSQ	0.129	0.036	0.063	0.203
SA → TSQ	Direct effects		−0.124	0.065	−0.252	0.005
	Indirect effects	SA-CPA-TSQ	0.117	0.033	0.061	0.189
SA → FSQ	Direct effects		0.145	0.056	0.035	0.255
	Indirect effects	SA-CPA-FSQ	0.100	0.028	0.051	0.159

Note: DA = deep acting, CP = customer participation, CPA = customer positive affect, SA = Surface acting, CI = consumer involvement, TSQ = technical service quality, FSQ = functional service quality.

**Table 6:** Tests for moderating effects.

Independent Variables		M-8	M-9
		CP	CPA
Control variables	Gender	0.130	0.113
	Age	0.078	−0.167 *
	Income	0.158 *	0.021
	Education	−0.151 *	−0.071
DA		0.363 ***	--
SA		--	0.494 ***
CI		−0.404 **	0.559 ***
DA * CI		0.216 ***	--
SA * CI		--	−0.062 *
R^2^/ΔR^2^		0.311 ***/0.136 ***	0.235 ***/0.011 *

Note: DA = deep acting, CP = customer participation, CPA = customer positive affect, SA = Surface acting, CI = consumer involvement. * *p* < 0.05; ** *p* < 0.01; *** *p* < 0.001.

**Table 7:** Test of conditional indirect effects.

Path		Effect	SE	95% Confidence Interval	
				Lower	Upper
DA→CP→TSQ					
CI	Low(−1SD)	0.021	0.037	−0.055	0.306
	High(+1SD)	0.345	0.064	0.224	0.477
DA→CP→FSQ					
CI	Low(−1SD)	0.007	0.015	−0.019	0.042
	High(+1SD)	0.122	0.047	0.037	0.219
SA→CPA→TSQ					
CI	Low(−1SD)	0.145	0.040	0.074	0.229
	High(+1SD)	0.058	0.039	−0.014	0.137
SA→CPA→FSQ					
CI	Low(−1SD)	0.124	0.031	0.066	0.189
	High(+1SD)	0.050	0.033	−0.010	0.120

## Data Availability

The data of this study are available from the corresponding author upon reasonable request.

## Appendix A

**Table A1:** The measurement scales.

Variables	Items	Developed by
Deep acting	Make an effort to actually feel the emotions that I need to display to customers. Really try to feel the emotions I have to show as part of my job. Try to actually experience the emotions that I must show.	Brotheridge and Lee (2003)
Surface acting	Resist expressing my true feelings. Pretend to have emotions that I don’t really have. Hide my true feelings about a situation.	Brotheridge and Lee (2003)
Customer participation	I spent a lot of time sharing information about my needs and opinions with the staff during the service process. I put a lot of effort into expressing my personal needs to the staff during the service process. I always provide suggestions to the staff for improving the service outcome. I have a high level of participation in the service process. I am very much involved in deciding how the services should be provided.	Chan et al. (2010)
Customer positive affect	I felt elated during the service. I felt preppy during the service. I felt enthusiastic during the service. I felt excited during the service. I felt strong during the service. I felt active during the service.	Pugh (2001)
Technical service quality	The employee is very capable of doing his/her job. The employee is highly trained in his or her specialty. The employee quite skilled in his/her job.	Gallan et al. (2013)
Functional service quality	The employee treated me with respect. The employee provided courteous and friendly service to me. The employee was considerate of my needs.	Gallan et al. (2013)
Customer involvement	My banking service with this bank is very important to me. My banking service with this bank is continually of interest to me. My banking service with this bank has a great concern with me. I am highly involved in reading information about banking services	M. F. Y. Cheung and To (2011)
